# Healthcare access among migrants in Morocco: perspectives of migrant communities, primary healthcare professionals and civil society actors

**DOI:** 10.1136/bmjgh-2025-018980

**Published:** 2025-12-17

**Authors:** Oumnia Bouaddi, Stella Evangelidou, Moudrike Abdellatifi, Farah Seedat, Wafa Chemao-Elfihri, Bouchra Assarag, Anna Deal, Hassan Chrifi, Nelly Chavassieux, Ibrahim M Sorie Turay, Cédric Kané Gohi, Tarik Oufkir, Ana Requena-Méndez, Sally Hargreaves, Mohamed Khalis, On behalf of the MENA Migrant Health Working Group, Asad Adam

**Affiliations:** 1 Mohammed VI International School of Public Health, Mohammed VI University of Sciences and Health, Casablanca, Morocco; 2 Faculty of Medicine, University of Barcelona, Spain; 3 Department of Public Health and Clinical Research, Mohammed VI Center for Research and Innovation, Rabat, Morocco; 4 Barcelona Institute for Global Health (ISGlobal, Hospital Clínic-Universitat de Barcelona), Barcelona, Spain; 5 The Migrant Health Research Group, Institute for Infection and Immunity, City St George's, University of London, SW17 0RE, London, UK; 6 National School of Public Health, Rabat, Morocco; 7 Maroc Solidarité Médico-Sociale, Rabat, Morocco; 8 Department of Medicine, Karolinska Institute, Stockholm, Sweden; 9 CIBERINFEC, ISCIII - CIBER de Enfermedades Infecciosas, Instituto de Salud Carlos III, Centro de Investigación Biomédica en Red de Enfermedades Infecciosas, Madrid, Spain; 10 Higher Institute of Nursing Professions and Health Techniques, Ministry of Health and Social Protection, Rabat, Morocco

**Keywords:** Health Services Accessibility, Qualitative study, Health services research, Global Health

## Abstract

**Introduction:**

Morocco’s position at the crossroads of Africa and Europe has made it a major transit and destination country for migrants. While migrants are entitled to free emergency and primary healthcare services, some challenges persist. This study aimed to explore the experiences of migrants in accessing healthcare services and to identify recommendations for improvement.

**Methods:**

This multisite qualitative study was conducted across five cities in Morocco between May 2023 and January 2024. Data were collected through semi-structured interviews and focus group discussions with 34 migrants, 17 migrant community leaders, 5 representatives of civil society organisations (CSOs), and 8 healthcare professionals. Migrants were recruited with the support of a Moroccan CSO, and primary healthcare professionals were recruited in health centres. Data were analysed using a hybrid thematic analysis approach, guided by Levesque’s Patient-Centered Access to Care framework.

**Results:**

We found that fear of costs, negative perceptions about the healthcare system, misconceptions about entitlement to services, cultural norms and health beliefs influenced participants’ health-seeking behaviours. Most reported free and easy access to primary healthcare, but administrative barriers, language challenges and medication costs persisted despite entitlement. Some migrant participants showed limited understanding of care pathways, leading to delays in care-seeking and fear of service denial—especially in the absence of peer accompaniment. Financial and administrative barriers were greatest at higher levels of care, posing challenges for uninsured migrants who formed the majority of participants. CSOs provided important support services but faced limits due to inconsistent funding and heavy centralisation.

**Conclusion:**

Morocco has become a global and regional champion in migrant health, through major policy and programmatic efforts. Yet, economic and sociocultural barriers still limit full service utilisation. Ongoing national reforms offer a chance to leapfrog towards universal health coverage through innovative migrant-inclusive health insurance schemes and empowered community actors.

**WHAT IS ALREADY KNOWN ON THIS TOPIC**

- Morocco, strategically located between Africa and Europe, has become a major transit and destination country for migrants and refugees.
- A pioneer in migrant integration, Morocco has made significant strides in providing migrants with equitable access to healthcare.

**WHAT THIS STUDY ADDS**

- By documenting migrant healthcare access from the perspectives of communities and multiple actors involved, this study addresses a key gap in the global literature on health and migration, particularly in the context of lower-middle-income receiving countries. While much of the existing evidence comes from high-income settings, this research offers important insights into how migrants experience healthcare and what practical challenges persist on the ground in a relatively new destination country like Morocco, which is currently undertaking major health reforms and making strides towards universal health coverage.

**HOW THIS STUDY MIGHT AFFECT RESEARCH, PRACTICE OR POLICY**

- This research contributes to a broader understanding of the successes and challenges facing health systems in lower middle-income receiving countries and in particular countries situated along major migratory corridors in the Global South, as they work to build inclusive models of care amid evolving demographic, policy and institutional landscapes.

## Introduction

 The number of international migrants has increased over the past decades, reaching more than 280 million globally in 2020.^1^ Some migrant groups experience precarious living and working conditions in host countries,^2 3^ leading to poor health outcomes.^4 5^ The World Health Organization (WHO) calls for universal access to healthcare for all population groups equitably.^4 6^ However, migrants continue to face well-documented barriers to healthcare.^4 7^

Morocco’s geographic position at the crossroads between Africa and Europe has made it a major transit and destination country.^8^ In 2020, Morocco hosted over 102 000 international migrants, half of which were female.^1^ As of 2025, the country also reportedly hosts more than 18 900 forced migrants from 64 countries, mainly Syria, Yemen and Sudan.^9^ National studies and reports indicate that improving quality of life and economic conditions, escaping conflict and political instability and crossing over to Europe are the major reasons for migrating to Morocco.^10^,^12^ The number of undocumented migrants is difficult to estimate, but existing estimates suggest that nearly half of migrants in Morocco are in irregular administrative situations, with most coming from Sub-Saharan African countries such as Guinea, Nigeria, Mali, Côte d’Ivoire and Cameroon, and more recent arrivals from Sudan and Yemen.^13^

Major advances were made by the government such as granting access for all migrants, regardless of their administrative or legal status, to free-of-charge emergency and primary care services across the country.^14 15^ This includes a package of preventive services (such as immunisation), prenatal care, hypertension and diabetes management as well as access to free essential medicines at primary health centres, on the same basis as Moroccan citizens.^14^ Likewise, migrants are included in national vertical programmes, such as the National Immunization Program, the National Programs for TB and HIV/AIDS among others.^14^ Efforts to ensure equitable access to health have culminated in the development of the National Strategic Plan for Health and Immigration spanning the period 2021–2025, which is the guiding instrument for the health of migrants in Morocco.^16^ Hospital regulations also mandate non-discriminatory access and explicitly state that payment is not a condition for admission,^17^ however, accessing secondary and tertiary care is often contingent on the ability to pay certain fees or enrolment in existing health insurance schemes. These include Régime d’Assistance Médicale (RAMED), the health insurance programme for economically disadvantaged populations, to which regular migrants and refugees are theoretically entitled^18^; and Assurance Médicale Obligatoire (AMO), which covers formal public-sector and private-sector employees as well as students, including international students. In 2022, these schemes were merged under AMO-Tadamon, a unified system designed to also integrate informal workers and low-income populations, allowing more equitable access to both public and private healthcare facilities.^19^

Despite their entitlement to services, existing but limited data suggests that seeking and utilisation of existing services by some migrant groups may be suboptimal due to a range of contextual and structural barriers.^14 16^ For example, one study showed that Sub-Saharan migrant children under 5 had low completion rates for routine immunisation (despite it being offered free of charge in primary care facilities), which was linked to persisting administrative and language difficulties.^20^

Current literature on migrant health in Morocco has mainly focused on the burden of disease,^21^ particularly sexual and reproductive health including HIV/AIDS^11 12 22 23^ and gender-based violence.^11 22 23^ Much of this research is based on quantitative surveys, leaving a significant gap in qualitative data that explores their experiences within the Moroccan healthcare system. In this study, we seek to understand the experiences of migrants in healthcare access across the levels of care through the perspectives of migrants and actors closely engaged with them, and to identify strategies for improving access and quality of care. This study provides important evidence for policymakers in Morocco by highlighting key bottlenecks to target within ongoing Universal Health Coverage (UHC) efforts and health system reforms, and aligning with global commitments like the Sustainable Development Goals and the Global Action Plan for migrant health.^15 24^

## Methods

The study is reported using the Standards for Reporting Qualitative Research.^25^ For the purposes of this research, we define a migrant as a foreign-born person who has been living in Morocco for more than 6 months, regardless of (1) their legal status, (2) whether the movement was voluntary or involuntary or (3) the reasons for moving. This definition considers the heterogeneity of migrant groups in Morocco including regular and irregular migrants and forcibly displaced populations (refugees and asylum seekers).

### Study design and setting

We conducted a multisite qualitative study in five cities: Rabat, Casablanca, Tangier, Oujda and Agadir; selected for their large migrant populations and the availability of organisations to support participant recruitment.

We used Levesque’s Patient-Centered Access to Care Framework^26^ to guide data collection and initial analysis. The framework addresses both organisational and individual barriers to care, outlining five dimensions of service accessibility (approachability, acceptability, availability and accommodation, affordability and appropriateness) and five corresponding patient abilities (to perceive, seek, reach, pay and engage). It has been widely applied to assess healthcare access globally, including in studies among migrants and ethnic minorities.^26^

### Study population and participant recruitment

We included four population groups. First, we included adult migrants aged 18 and over, regardless of age, gender, legal status and duration of stay in the country. Second, we interviewed migrant community leaders (adult migrants supporting local community-based organisations (CBOs) or civil-society organisations (CSOs) in social and health activities, and who are either formally employed by these organisations, working on a volunteer basis, or engaged on a project basis). Third, we interviewed representatives of CSOs/CBOs and primary healthcare professionals. Migrant community leaders, primary healthcare professionals and civil society representatives were included to understand their perspectives on migrants’ experiences, as proxies. All migrant participants and CSO representatives were purposively recruited with support from a Moroccan CSO that provides services to vulnerable migrant populations in Rabat. This organisation served as the main partner for the study and helped identify other local migrant-supporting organisations to assist with recruitment in the remaining data collection sites. Primary healthcare professionals were recruited in primary healthcare facilities in Rabat with support from the directors of primary care centres and the research team in Morocco.

### Data collection

First, we did in-person focus group discussions (FGD) with adult migrants and semi-structured interviews with migrant community leaders. The constitution of FG was done based on key characteristics such as gender and language spoken. Second, we did in-person semi-structured interviews with primary healthcare professionals and FGDs with representatives of CSOs supporting migrants. We conducted individual interviews with health professionals due to their limited availability and the difficulty of bringing them together for focus groups during working hours. CSO representatives were all affiliated with associations based in the same region (Rabat-Casablanca), which made FGDs feasible and practical. For migrant participants (other than community leaders), we chose the FGD format to better understand collective experiences and encourage dialogue and shared learning. Based on previous research experience with migrants in Morocco, we have learnt that these activities also offer a valuable opportunity for information exchange, particularly in groups where some participants have lived in the country longer or have had more interactions with the health system.

All activities were conducted in-person between May 2023 and January 2024, often in the morning between 9:00 and 15:00 at the premises of CSOs. All activities were done in French, English, Arabic or Moroccan Dialect (Darija) by one researcher (OB) while one observer (MA) took field notes (eg, non-verbal cues, group dynamic) using an observation grid. We used four different topic guides for each participant profile, which were developed iteratively by the research team. The topic guides focused on questions related to social needs related to health, health needs, views around the healthcare system, experiences in seeking, reaching and using services and recommendations. Each interview lasted 25–40 min and each FGD lasted 50–90 mins. For participants speaking only their country of origin’s local dialect, a community leader joined the session and provided real-time interpretation; however, this was only the case for one participant during an FGD. Data collection ended when no new concepts were emerging and when data saturation was reached. All migrant participants were provided with refreshments during the activities and reimbursed for transportation costs.

### Data analysis

All sessions were audio-recorded and transcribed verbatim using Nvivo V.1.7. transcription module or manually for transcripts in Moroccan Darija. Transcripts were verified for accuracy and completeness before the analysis and translated to English by the first author. For both individual interviews and FGD, we adopted a hybrid thematic analysis approach, which combines both deductive, top-down and theory-driven reasoning described by Crabtree and Miller (1999) as well as inductive, bottom-up and data-driven process outlined by Boyatzis.^27 28^ For the deductive element, the a priori framework, Levesque’s model domains and subdomains were set up as themes and subthemes into NVivo. The lead researcher (OB) thoroughly read and familiarised herself with the data and generated initial codes to fit under the themes in the model. In parallel, an inductive process was applied to allow for new themes and codes to emerge from the data. A codebook was generated and revised repeatedly and several rounds of discussion between OB and SE around the accuracy of the codes, researcher triangulation and fidelity to participants’ meaning, until consensus was reached on the final codebook.

### Reflexivity and positionality

We acknowledge the power dynamics between researchers and participants. The lead researcher (OB), a Moroccan female with a clinical and public health background, may have been perceived as an authority figure, which may have shaped what participants shared or withheld. To mitigate this, we collaborated with CBOs and local leaders to build trust and facilitate recruitment, and all data collection took place in familiar settings using languages spoken by participants. During analysis, we practised self-reflexivity and regularly discussed codes and themes between researchers with different backgrounds to minimise bias.

### Patient and public involvement

Interviews and FGDs were conducted by the research team, with support from community leaders to facilitate data collection, build trust and provide clarifications and explanations at the start of interviews as well as interpretation where needed. No direct interaction with the general public took place.

## Results

### Participant demographics

We included 65 participants: 17 migrant community leaders, 34 migrants in five FGDs, six CSO representatives in one FGD and 8 primary care professionals (table 1). Among community leaders, 12/17 were male, mean age 37.4 years (SD±7.5), with an average 9.4 years in Morocco (SD±4.9). Among migrant participants, 33/34 were female, mean age of 29.0 years (SD±8.0), with similar length of stay. More migrants in FGDs were undocumented (15/34) compared with community leaders (7/17 with residence permits). All participants were from the WHO African Region, mainly from Ivory Coast (n=18) and Guinea (n=9).

**Table 1:** Characteristics of the study population

Characteristics	Migrant community leaders (N=17)	Migrants (N=34)
Migrants and migrant community leaders		
Age in years, mean (SD)	37.4±7.5	29.0±8.03
Sex		
Men	12 (70.6)	1 (2.9)
Women	5 (29.4)	33 (97.1)
Site		
Rabat	5 (29.4)	6 (17.6)
Tangier	4 (23.5)	10 (29.4)
Oujda	2 (11.8)	9 (26.5)
Agadir	5 (29.4)	9 (26.5)
Casablanca	1 (5.9)	–
Time of stay in Morocco in years (mean±SD)	9.35±4.91	4.22±3.85
Less than 5 years	2 (11.8)	22 (66.7)
5–9 years	7 (41.2)	9 (27.3)
10 years or more	8 (47.1)	2 (6.1)
Education		
None	–	3 (8.8)
Primary	2 (12.5)	4 (11.8)
Secondary	6 (37.5)	13 (38.2)
High school	1 (6.3)	4 (14.7)
Higher education	5 (31.3)	6 (17.6)
Prefer not to say	2 (12.5)	3 (8.8)
Country of origin		
Ivory Coast	4 (23.5)	14 (41.2)
Guinea	2 (11.8)	7 (20.6)
Cameroun	3 (17.6)	4 (11.8)
Congo-Brazzaville	1 (5.9)	3 (8.8)
Democratic Republic of the Congo (DRC)	–	2 (5.9)
Central African Republic	1 (5.9)	1 (2.9)
Burkina Faso	1 (5.9)	1 (2.9)
Mali	–	1 (2.9)
Nigeria	3 (17.6)	1 (2.9)
Senegal	1 (5.9)	–
Liberia	1 (5.9)	–
Employment status		
Employed	12 (70.6)	13 (38.2)
Unemployed	5 (29.4)	19 (55.9)
Prefer not to say	–	2 (5.9)
Administrative status		
Undocumented*	5 (29.4)	13 (27.6)
Asylum seeker	–	2 (5.9)
Refugee	–	4 (11.8)
Documented (has a resident permit)	7 (41.2)	5 (14.7)
Prefer not to say	5 (29.4)	10 (29.4)

Health professionals and CSO representatives		
Characteristics	Healthcare professionals (N=8)	CSO representatives (N=6)
Age in years, mean (SD)	41.4±7.95	45.6±6.09
Sex		
Men	1 (12.5)	1 (20.0)
Women	7 (87.5)	5 (80.0
Country of origin		
Democratic Republic of the Congo (DRC)	–	3 (60.0)
Ivory Coast	–	1 (20.0)
Italy	–	1 (20.0)
Morocco	8 (100)	–
Prefer not to say	–	1

* Includes participants who still possess a valid or expired home country passport but do not have a residence permit.

CSO, civil society organisation.

### Healthcare seeking

**Figure 1:**
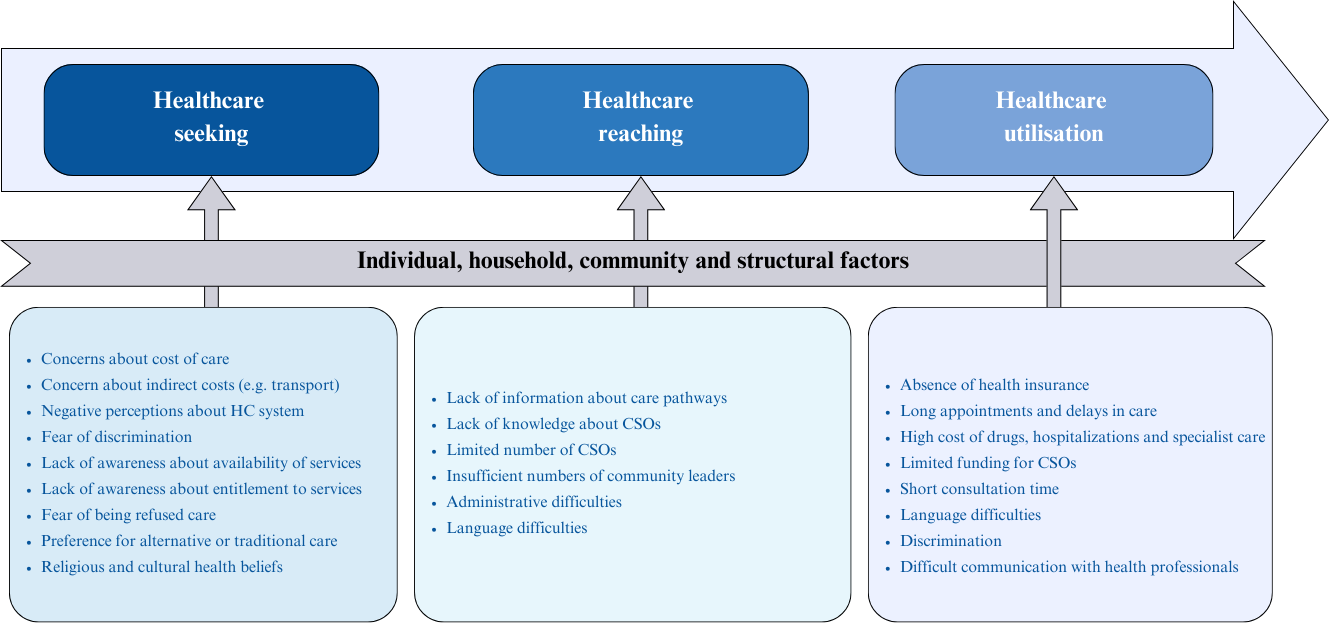
Challenges in seeking, reaching and utilising healthcare services by migrants. Adapted from Levesque’s Conceptual Framework of Access to Health, 2013.^22^ CSO: Civil Society Organisations; HC: Healthcare

Participant narratives revealed some challenges impeding care seeking (see figure 1). These included concerns about direct and indirect costs (mainly cost of drugs and transportation), negative perceptions about the healthcare system, fear of discrimination and lack of knowledge about entitlement to and the availability of free services for migrants. Additionally, those who were not in a regular administrative situation were reported to avoid seeking care due to fear of being sent away:

There are already a lot of cases, a lot of people have [health] problems, but they don't know where to go. Male community leader, Rabat

They are reluctant to go to the health structure because they are concerned about the costs. Female community leader, Rabat

Participants also reported health beliefs rooted in religious and cultural norms were also reported by participants and thus affecting their health-seeking behaviours. For instance, one participant, who identified as Christian and reported struggling with mental health issues, did not seek care, believing it was something she could overcome on her own:

I'm a Christian, I'm a believer. I prayed […] that’s when I started to heal. Because I felt it was a battle I had to fight on my own. Female migrant, Tangier

Participants also reported beliefs related to multiple specific diseases or health interventions, which affected health-seeking behaviour. These included beliefs about potential harms related to vaccination (both for children and specific vaccines such as COVID-19) and perceived stigma related to mental health rooted in cultural norms in participants’ countries of origin. For example, one participant described how, in their country of origin, the common response to psychological distress was to rely on close social networks or spiritual practices, rather than seeking professional help. They emphasised that mental health issues were not widely acknowledged or discussed, and formal psychological support was largely absent or unfamiliar:

Back home in Africa, we don't really know much about this stuff [mental health issues]. Maybe when your problems get out of hand, you have to go and see a psychologist? No, that doesn't exist in sub-Saharan Africa […] You talk to each other, it makes you forget. Female community leader, Rabat

As an alternative to mainstream services, participants described turning to traditional remedies and practices, often accessed through their social networks. These included purchasing specific leaves, potions and traditional medicines used to treat a range of health symptoms as well as for women’s intimate hygiene. This reliance on traditional care was seen as more accessible, familiar and more culturally resonant than formal healthcare services:

We prefer to go out and buy potions and traditional leaves. Female migrant, Tangier

### Healthcare reaching

Some participants described a lack of clear information and guidance on how to navigate the care pathway. This often resulted in confusion, mistrust and the mistaken belief that services were being denied. One community leader emphasised that not knowing how the system works and about existing support services, such as social assistants in hospitals, can lead people to avoid care altogether out of fear of costs or rejection:

Imagine a woman giving birth in a hospital, you don't have a penny, and you don't know the government system. In Morocco, there is a system of assistance, called social assistant. But if you don't know about it and you're afraid to go to the hospital, you'll give birth at home. Male community leader, Rabat

Language difficulties were also reported by migrant community leaders and CSO representatives, particularly among English-speaking migrants. In addition, community leaders reported that the situation is more challenging for migrants who only speak their local countries’ dialects:

There is also a problem with migrants who do not speak French, nor English, and who speak their local dialect. They also have health problems. Female migrant, Oujda

Migrant participants emphasised that being accompanied to healthcare services by community leaders was a key strategy to overcome challenges related to limited knowledge of the healthcare system and language barriers. This was seen as especially important for newly arrived migrants, who often face uncertainty, fear and practical difficulties when trying to access care, and who are often unsure about their entitlement to care. Community leaders who had experience navigating the system were viewed as trusted intermediaries who could provide support with administrative procedures, explain care pathways and facilitate communication between patients and providers:

I mainly support women during childbirth for paperwork, so I gained a lot of experience there. There is a communication problem […] these women do not know, do not know the process. Female migrant, Agadir

If there are not community agents who will assist them at the hospital, they cannot communicate with the doctor. Female migrant, Oujda

Despite the success of accompaniment in bridging the gap between migrants’ entitlements and their actual use of services, participants highlighted that this approach remains limited in scale. Many community agents operate on a volunteer or project-funded basis, and their numbers were seen as insufficient to meet the growing needs of migrant communities, particularly in underserved regions. Civil society actors and community leaders highlighted that the burden placed on a small pool of intermediaries is unsustainable and that distribution of migrant supporting organisations is heavily centralised leaving regional gaps:

Those [migrants] from Casa also demand [CSO services] because we need to decentralize things. We cannot only be focused on Rabat. CSO representative, Rabat

While all migrant participants confirmed that access to primary care facilities was indeed not conditioned on having a regular administrative status as per existing policies; some administrative issues were still described in practice. For example, not having a fixed home address presented a barrier to accessing health services due to the health system’s organisation by geographic catchment areas. This means that health centres serve specific neighbourhoods, and patients are typically expected to access care at the centre covering their place of residence. This was not always well understood by migrant participants, and being asked to provide proof of residence was often misunderstood as being turned away. However, the type of proof required sometimes posed an issue. For example, participants explained that a rent contract may be difficult to obtain if a migrant rents accommodation that is shared by multiple people, which participants described as common in major cities. In such cases, alternative documents were accepted, such as a utility bill or a written statement from the landlord confirming residence in the area. Community leaders noted that healthcare professionals at the primary care level were generally understanding and more flexible on this matter. Likewise, CSO representatives emphasised that accompaniment by a trusted intermediary helped migrant patients better navigate these challenges:

The main concern is the place of residence because each health center covers a pre-determined catchment area […] However, if someone from an association accompanies the person and explains the situation, it is usually resolved. CSO representative, Rabat

For secondary and tertiary care services, reaching healthcare services was reported to be extremely challenging. Migrant community leaders described that, outside emergency services, having an identity document was often required to access from secondary and tertiary care services including hospitalisations, diagnostic and laboratory services; which made these services largely inaccessible to undocumented migrants compared with those with a residence permit or a refugee status:

At the secondary level, it is totally different because you need an identity document, at least a passport or an asylum application. You need a document. CSO representative, Rabat

### Healthcare utilisation

At the point of care, migrant participants reported delays and long waiting lists for specialist consultations, noting that this was also experienced by Moroccan citizens, particularly at higher levels of care. Out-of-pocket expenses for medications were reported across all levels of the health system, while costs related to diagnostic tests, specialist care and hospitalisations were particularly burdensome at the secondary and tertiary levels. Both migrants and health professionals emphasised that these expenses posed a major barrier, especially for uninsured and undocumented migrants which made up a majority of participants in this study:

We are really in need and in terms of medications. Sometimes they prescribe medications, but maybe the person does not have money for the medications. Male community leader, Agadir

Migrant community leaders described the role of CSOs in supporting the provision of medication to migrants. However, project-based funding for CSOs, which often led to a cessation of support once the funding or project period ended, was seen as a major caveat. It was also mentioned that, although some CSOs covered medication costs, treatments like vitamins and supplements were often not included, and the list of prescribed drugs was not always completely provided, sometimes leading to migrant patients not pursuing the full treatment course prescribed to them:

We go there [to the association] to get free medication, but sometimes they say there is no medicine, and we have to buy it at the pharmacy. Other times, they give us medicine, but if it’s not on their approved list, the association does not cover it. Female migrant, Oujda

Other challenges affecting healthcare utilisation included difficulties in communication between migrants and healthcare providers. These challenges stemmed from a combination of language barriers, limited consultation time and, in some cases, perceived discriminatory attitudes. Migrant participants reported feeling that their health concerns were not always taken seriously, with some expressing frustration at not receiving proper clinical attention during consultations:

They [health professionals] should not just ask questions, they should examine the patient. As I tell you, even friends who go to consult tell me they don’t. Male community leader, Oujda

### Recommendations for strengthening access to healthcare

Participants highlighted several strategies to improve healthcare access and quality for migrants (box 1). Expanding health insurance schemes to include undocumented migrants was emphasised to reduce out-of-pocket costs and support access to specialist care, medications, diagnostics and hospitalisation:

Insurance, insurance, we need to find a solution for them. Female family physician, Rabat

Box 1 **Grassroots recommendations to strengthen access to healthcare for migrants in Morocco**

**Increase information, orientation and outreach**

- Improve knowledge about entitlement to healthcare services, particularly for newly arrived migrants, to ensure they are informed about available resources and support and to address fear and misconceptions about being denied care.
- Increase timely information on the pathways to care and the organisation of the healthcare system among migrant groups regardless of legal status in order to manage expectations around service availability and any administrative requirements.

**Develop appropriate health insurance schemes**

- Expand health insurance schemes to include undocumented migrants to support accessing specialist care, medications, diagnostic and increase financial protection for all migrant groups regardless of legal status.

**Strengthen the role and engagement of community leaders**

- Increase the number of community leaders to assist migrants in navigating healthcare services.
- Support diverse groups of community leaders in order to support various migrant groups to overcome language, communication and cultural barriers.

**Increase support for migrant-supporting organisations as partners to public health facilities**

- Secure sustainable funding for migrant-supporting organisation to ensure they can continue providing essential accompaniment and support services to migrants.
- Support the creation of migrant-supporting organisations in non-major urban areas to provide outreach services in collaboration with public health facilities.

**Improve coordination between healthcare system and community actors**

- Increase coordination between the healthcare system and community actors and streamline communication between them.

**Increase training of health professionals**

- Increase the implementation of training programmes to increase awareness about the specific needs of migrants and provide up-to-date knowledge on fast-evolving health policy reforms related to migrant entitlements.

**Address social determinants of health**

- Continue to address broader social determinants of health including social and economic factors (e.g, adequate housing, employment).

Strengthening accompaniment and the role of community leaders was also recommended, with increased numbers and diversity of leaders to help migrants navigate health services, overcome language and cultural barriers and provide peer support:

We need people to accompany us […] in hospitals and health structures. Female migrant, Agadir

Improving coordination between healthcare facilities and community actors was suggested to streamline communication, ensure timely referrals and increase awareness of available services. Participants also stressed the need for sustainable funding for migrant-supporting organisations, particularly in under-served regions, to maintain outreach and support activities. Increasing information and orientation about entitlements and healthcare pathways was recommended to address misconceptions and fears, alongside continuous training for health professionals on needs and entitlements of migrants. Finally, sustainable efforts to address social determinants of health, such as employment and housing, were recommended as they affect migrants’ abilities to pay for services and prioritise their health:

Migrants need jobs, not just aid. Male community leader, Agadir

## Discussion

This study addresses a knowledge gap in the Global South by examining migrant healthcare access in a lower middle-income and relatively new destination country. We found that fear of costs, misconceptions about entitlements, cultural norms and health beliefs shaped health-seeking behaviours. While most participants reported free access to primary care, barriers included administrative procedures, language difficulties and out of pocket spending on medication. Limited knowledge of the health system, especially regarding care pathways for different migrant groups, led to delays and fear of being denied care. Access challenges were greatest at higher levels of care, where documentation and payment were often required, particularly affecting uninsured migrants. Although CSOs provided outreach, accompaniment, language support and some healthcare cost coverage, their impact was constrained by limited funding and heavy centralisation. These findings highlight that migrants experience specific barriers unique to their status, while also facing systemic issues related to the existing challenges in the healthcare system.

In this study, participants reported multiple reasons for not seeking care. Likewise, studies among migrants in both high and middle-income settings have shown that a lack of awareness of one’s right to healthcare and uncertainties about navigating the healthcare system are significant barriers to seeking care.^29 30^ Several studies globally have also shown that this leads some migrants to adopt alternative health-seeking strategies, such as self-medication or the use of traditional remedies, which is consistent with our findings.^29^ Similarly, low levels of health literacy often deter migrants from seeking healthcare and following health-related instructions.^4^ In Morocco, a survey of 1700 migrants showed that less than half were aware of their entitlement to free services in primary care centres.^31^ For undocumented migrants, the literature shows that these deterring factors are compounded by the fear of deportation.^29 32^ However, appropriate strategies—such as linguistic support, cultural sensitivity and consideration of the specific needs of these populations—have been proven effective in dissipating these concerns.^4^

Another key finding of this study is the emphasis on the high cost of essential medications and specialist care, and the inability to pay for secondary and tertiary healthcare services. This is likely explained by the high number of undocumented migrant participants in this study, who are not eligible to existing insurance schemes and are therefore more vulnerable to financial hardship when seeking higher levels of care. This is consistent with the literature in various settings where the cost of drugs and associated out-of-pocket spending significantly impacts access to care for migrants, even for relatively better-served groups such as refugees where services are subsidised by the government and international agencies.^33 34^ Often, this challenge also affects the host populations in the absence of universal insurance schemes. For instance, a study among mainly insured Moroccans showed that 89% perceived the price of medicines as a significant barrier to care.^35^ However, the host population typically has access to compulsory medical insurance. Even at the primary care level, where essential medicines are officially free for all migrants, participants still reported out-of-pocket payments. This may be due to migrants not being accounted for in medicine and vaccine planning, leading to undersupplied facilities in high-migrant areas.^36^ While some associations cover certain costs, CSO representatives reported limited capacity, likely worsened by reduced global health funding.^37^

The prohibitively high cost of secondary and tertiary care also remains a major barrier for undocumented and therefore uninsured migrants in this study, as also shown in broader literature.^4 38^ Globally, several financing strategies have been used to increase financial protection for migrants, including free health facilities, medical aid schemes and solidarity funds.^39^ In Morocco, recent stakeholder consultations on new financial governance models for migrant health showed support for multicomponent frameworks combining the expansion of compulsory health insurance to all migrants, leveraging public–private partnerships and establishing a national solidarity fund for migrant health.^40^ The 2022 Framework Law on social protection offers a timely opportunity and a major stepping stone to building such model.^19^ Currently, much of the gap in care provision is filled by NGOs and CSOs, but this approach is limited by sporadic funding and centralised operations in urban centres as stressed by our study participants. Formal and structural partnerships with government institutions are needed for better knowledge sharing and coordination, which was reported to be insufficient by health professionals in this study.^41^ Another potentially immediate step towards achieving financial protection for some migrant groups is the effective implementation and operationalisation of their inclusion in the RAMED scheme. In 2015, a framework agreement between four ministries was signed to provide regular migrants and refugees with access to a healthcare package equivalent to that offered to economically disadvantaged Moroccan citizens.^18^ However, nearly a decade later, the operationalisation of this policy remains unclear with research suggesting that both healthcare providers and eligible migrants may be unaware of the scheme or the procedures required to access it.^36^ Finally, context-appropriate forms of Community-Based Health Insurance models, which pool community resources to improve access for low-income populations, could be explored, as they have shown promise in increasing service utilisation, although the evidence around their ability to ensure financial risk protection is mixed, and concerns remain around their sustainability due to inherent adverse selection.^42 43^

This study has some limitations. First, the areas studied are well-serviced, with healthcare facilities and CSOs. Migrants in underserved regions may have differential experiences and health needs, which should be explored in future studies. Second, migrant men were under-represented in the FGDs, likely because the organisations supporting data collection mainly served women. In contrast, most community leaders were male, which may reflect women’s limited empowerment to take on such roles, possibly due to caregiving responsibilities. The predominance of male community leaders may also be linked to convenience sampling, as two male individuals supported recruitment in three of the five sites. Furthermore, the more secure legal status of community leaders may be attributed to the nature of these roles (often voluntary or project-based), which are less accessible to undocumented migrants struggling to meet basic needs. Finally, while this study focuses on barriers from the migrant perspective, it is important to acknowledge provider-level barriers across different levels of care, which have not been examined in this study.

This research has practical implications for informing policies and interventions to address the health needs of migrants in Morocco. Accompaniment by peers should be a key strategy to support migrants, especially newcomers, in navigating the complexities of the healthcare system—a model used by CSOs in Morocco but one that needs to be expanded and made more accessible particularly in underserved regions and with more formal partnerships to facilitate knowledge sharing across different regions. The non-affordability of medicines and services at higher levels of care must be addressed through innovative financing schemes and the expansion of health insurance to include undocumented migrants who are more likely to incur catastrophic health expenditures. Finally, continuous intersectoral efforts to address the broader health-related social needs remain critical, as they are intricately linked to health outcomes, and without meeting basic social needs such as housing and employment, individuals often deprioritise their health, leading to adverse health outcomes.^44^

## Conclusions

Morocco has emerged as a global and regional champion in migrant health, making significant strides in expanding access to care for migrants through years of policy and programmatic efforts. Yet, persistent economic barriers and informal sociocultural obstacles continue to limit the full utilisation of healthcare services across different levels of care. Ongoing national reforms and Morocco’s health equity framework present a critical opportunity to leapfrog towards a more inclusive and equitable health system. Achieving this will require stronger coordination with community actors, their meaningful empowerment and the development of migrant-inclusive health insurance schemes.

## Data Availability

Data are available upon reasonable request.
